# DNA G-quadruplex structures act as functional elements in α- and β-globin enhancers

**DOI:** 10.1186/s13059-025-03627-1

**Published:** 2025-06-04

**Authors:** Colm Doyle, Krzysztof Herka, Sean M. Flynn, Larry Melidis, Somdutta Dhir, Stefan Schoenfelder, David Tannahill, Shankar Balasubramanian

**Affiliations:** 1 Cancer Research UK Cambridge Institute, Li Ka Shing Centre, Robinson Way, Cambridge, CB2 0RE UK; 2 Epigenetics Programme, Babraham Institute, Cambridge, CB22 3AT UK; 3 Yusuf Hamied Department of Chemistry, University of Cambridge, Cambridge, CB2 1EW UK; 4 School of Clinical Medicine, University of Cambridge, Cambridge, CB2 0SP UK; 5 Present Address: Department of Clinical and Biomedical Sciences, Faculty of Health and Life Sciences, Royal Devon & Exeter Hospital, University of Exeter, RILD Building, Barrack Rd, Exeter, EX2 5DW UK

**Keywords:** Enhancers, G-quadruplex structures, 3D chromatin interactions, Epigenetics

## Abstract

**Background:**

Enhancer elements interact with target genes at a distance to modulate their expression, but the molecular details of enhancer–promoter interaction are incompletely understood. G-quadruplex DNA secondary structures (G4s) have recently been shown to co-occur with 3D chromatin interactions; however, the functional importance of G4s within enhancers remains unclear.

**Results:**

In this study, we identify novel G4 structures within two locus control regions at the human α- and β-globin loci. We find that mutating G4 motifs by genome editing prevents their folding into G4 structures in cells and disrupts 3D enhancer–promoter interactions and target gene expression in a manner comparable to whole enhancer deletion. Furthermore, restoration of G4 structure formation using a dissimilar G4-forming primary sequence recovers specific enhancer-gene interactions and gene expression. Through proteomic, biophysical, and genomic profiling, we find that enhancer G4s are tightly linked to the maintenance of an active chromatin state and RNA polymerase II recruitment to regulate target gene expression.

**Conclusions:**

Our study shows that folded G4 structures can act as functional elements that mediate 3D enhancer–promoter interactions to support enhancer-driven globin gene regulation.

**Supplementary Information:**

The online version contains supplementary material available at 10.1186/s13059-025-03627-1.

## Background

Accurate and faithful regulation of gene expression is largely controlled by the interactions between two genomic elements, the promoter and enhancer. Promoters define the positioning of RNA polymerase for the advent of transcription, and enhancers encode regulatory information to allow precise spatiotemporal control of gene transcription [1, 2]. How this regulatory information is encoded in enhancers remains a critical gap in our understanding of enhancer-driven gene regulation.

**Fig. 1:**
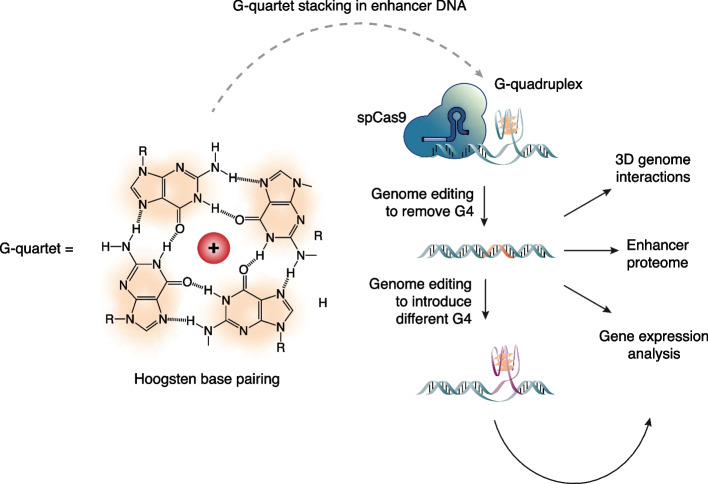
Study design to investigate folded G4 structure at enhancers. Four guanine nucleobases interact via Hoogsten-base-pairing to form a G-tetrad (orange). Stacking of G-tetrads forms G-quadruplex DNA secondary structures, which are prevalent at enhancers. The experimental design investigates endogenous G4 structure at enhancers in cells via CRISPR-Cas9 genome editing to remove or replace G4 structure

DNA G-quadruplex secondary structures (G4s) have emerged as key genome elements with causal effects on transcription [3–6]. G4s form in guanine-rich regions through guanine-guanine Hoogsteen base pairing to form G-tetrads, which assemble into stacks stabilized by cations to form a folded G4 structure [7] (Fig. 1A). G4s form in both endogenous promoters and enhancers [8–13], however the functional role of G4 s has only been comprehensively tested in promoters, where they co-ordinate nucleosome positioning [5] and influence the binding of many chromatin regulators [14–16] to positively affect transcription [6]. Thus, it is not known if G4s function in a similar manner at endogenous enhancers or if they contribute to the unique aspects of enhancer activity such as distal control of gene activation.

We set out to evaluate whether G4 folding is functionally important in the context of specific enhancers. For this, we focused on the paradigm of globin gene regulation, which has well-characterized 3D enhancer–promoter interactions [17–20]. Understanding globin gene regulation holds significant clinical relevance since expression imbalances in globin genes underlie many serious hemoglobinopathies, such as thalassemias [21]. In these disorders, perturbations in globin chain levels lead to red blood cell dysfunction and subsequent disease. The link between congenital globin disorders and genetic aberrations of their enhancers has helped to reveal fundamental concepts in enhancer biology and disease mechanisms [22, 23].

The sequential expression of α- and β-globin genes during development is dictated through 3D interactions between a series of enhancers found ~30–65 kb upstream of their globin target genes in locus control regions (LCRs) [22–29]. Individual enhancers also carry DNase I hypersensitivity sites (HSs) within sub-topologically associated domains (TADs) [30]. For the α-globin LCR, individual enhancers are thought to act as independent regulatory units [31], with HS-40 (also known as multispecies conserved regulatory sequence 2, MCS-R2) regulating ~50% of target gene expression [32]. In contrast, the β-globin regulatory landscape is characterized by multiple interdependent enhancers that regulate the expression of several β-like-globin genes [33], with HS2 and HS3 accounting for the majority of enhancer activity [34–36]. Crucially, the presence and potential function of a folded G4 in the key globin enhancers has not yet been considered.

Herein, we identify that the critical globin enhancers, HS-40 for the α-globin locus and in HS2 for the β-globin locus, each display a folded G4 structure in the chromatin of human K562 erythroleukemia cells. Using genetic substitutions to prevent G4 structure formation (Fig. 1), or by introducing alternative G4 structures, we demonstrate that the folded G4 structure within both globin enhancers is positively linked with 3D enhancer–promoter interactions and expression of α-globin (*HBA1*) and ε-globin (*HBE1*) genes. Furthermore, globin enhancer G4 s were found to be tightly associated with an active chromatin state. Taken together, our results provide robust support for G4 structures as functional elements in enhancer-driven globin gene regulation.

## Results

### Folded G4 structures form in α- and β-globin enhancers

**Fig. 2:**
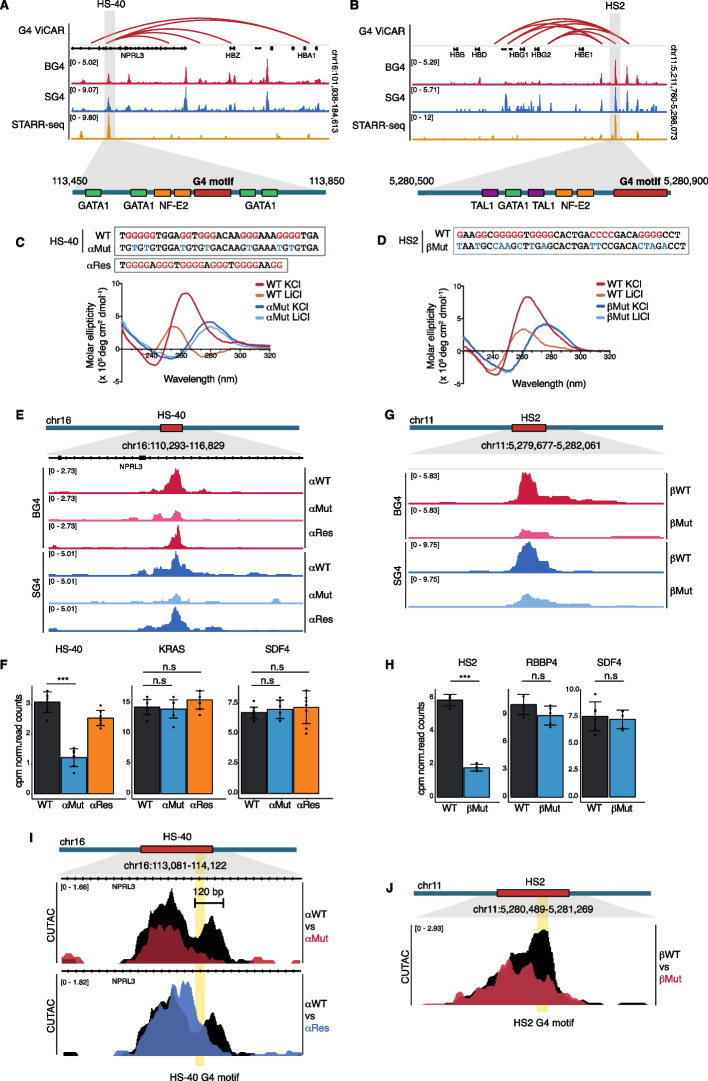
Genome editing of enhancer G4s reveal functional roles in maintenance of open chromatin.** A**–**B** Integrative Genomics Viewer (IGV) browser view of G4 structures using BG4 and SG4 G4-structure-specific antibodies by CUT&Tag across the α- and β-globin loci. Significant (*q* < 0.01) 3D interactions as determined by G4 ViCAR (GEO accession: GSE250219) are displayed above as red arcs. STARR-seq data from ENCODE indicates sequences with enhancer activity in reporter assays (www.encodeproject.org ENCSR858MPS). Gray vertical shading indicates enhancer regions with G4 signal in HS-40 and HS2 enhancers. A schematic below indicates the position of the G4 motif relative to transcription factor binding sites. **C**–**D** Circular dichroism spectra of G4-forming sequences for α-globin HS-40 (left) and β-globin HS2 (right) enhancers and their corresponding mutated sequences in the presence of KCl or LiCl. **E** IGV browser view of G4 signal for BG4 and SG4 CUT&Tag at the α-globin HS-40 enhancer in wild type (WT), G4 mutated (αMut) and G4 restored (αRes) cells. **F** Bar plots of BG4 counts per million (cpm) normalized read counts at the α-globin HS-40 enhancer and non-targeted promoter G4 sites, KRAS and SDF4 in wild type (WT), G4 mutated (αMut) and G4 restored (αRes) cells (*n* = 7) error bars represent the standard deviation (SD), ***P-value < 0.001, n.s is not significant. **G** IGV browser view of G4 signal for BG4 and SG4 CUT&Tag at the β-globin HS2 enhancer in wild type (WT) and G4 mutated (βMut) cells. **H** Bar plots of BG4 cpm normalized read counts at β-globin HS2 and non-targeted promoter G4 sites RBBP4 and SDF4 in wild type (WT) and G4 mutated (βMut) cells (*n* = 6) error bars present the SD, ***P-value < 0.001, n.s is not significant. **I** IGV browser view of CUTAC signal at the α-globin HS-40 enhancer, comparing wild type vs G4 mutated (αMut), top; or G4 restored cells (αRes), bottom. Yellow bar highlights the position of the HS-40 G4 sequence motif. Scale bar indicates a 120-bp CUTAC region affected by HS-40 G4 loss. **J** IGV browser view of CUTAC signal at the β-globin HS2 enhancer comparing wild type (WT) vs G4 mutated (βMut) cells. Yellow bar highlights the position of the HS2 G4 sequence motif. See also Additional file 1: Figs. S1–S4

We first established whether folded G4s form in key enhancers that regulate α- and β-globin gene expression. For this, we used human K562 erythroleukemia cells for which extensive epigenomic and 3D chromatin information is available [37]. We used two independently derived G4 structure-specific antibodies, BG4 [11] and SG4 [12], to detect sites of folded G4s in chromatin by CUT&Tag [38] within the globin LCRs. G4 structure formation was clearly observed within both the α-globin HS-40 and β-globin HS2 regions, both of which display enhancer activity in STARR-seq assays [39] in K562 cells [37] (www.encodeproject.org ENCSR858MPS; Fig. 2A, B). BG4 and SG4 also show some weaker binding to sites outside of those with strong enhancer activity (as defined by STARR-seq). While this suggests further G4 roles, the focus of our investigation was on G4s within regions with clear enhancer function. Moreover, using an extension of G4 CUT&Tag called ViCAR [40] that allows simultaneous mapping of 3D chromatin interactions at defined chromatin features, reveals that G4s in HS-40 and HS2 are folded during 3D enhancer–promoter interactions (Fig. 2A, B).

### Mutation disrupts G4 structure formation in α- and β-globin enhancers

We next aimed to assess if the presence of a folded G4 is required for HS-40 or HS2 enhancer activity. To test this, we used CRISPR-Cas9 editing to generate HS-40 or HS2 enhancers that lack a G4 structure.

First, we identified the G4 sequence motifs corresponding to the G4 peaks at both enhancers that contained at least four G_>2_ consecutive runs by visual inspection (Fig. 2C, D), and confirmed that these were the strongest G4 motifs predicted using G4Hunter [41]. The HS-40 G4 sequence is flanked by binding sites for the erythroid transcription factors (TFs) GATA1 and NF-E2 [42] (Fig. 2A), and the HS2 G4 sequence is positioned 60 bp from a cluster of GATA1, NF-E2, and TAL1 TF binding sites [43, 44] (Fig. 2B). Thus, both G4 sequences are independent of the binding sites for these TFs, which are critical for globin enhancer function.

To abolish G4-forming potential of the HS-40 and HS2 G4s, we designed G base mutations (7 for HS-40 and for 11 HS2) that interfere with Hoogsteen bonding essential for G-tetrad formation [45]. The resulting sequences are referred to as αMut and βMut (Fig. 2C, D). We sought to introduce as few mutations as possible to avoid inadvertent introduction of TF motifs while ensuring complete G4 disruption.

Next, we biophysically confirmed that the chosen mutations did indeed abrogate G4 folding. Circular dichroism (CD) spectra of oligonucleotides for wild type (WT) HS-40 and HS2 G4 displayed characteristic signatures of parallel G4 structure formation [46] in KCl (maxima ~260 nm and minima ~240 nm) that were reduced under less stabilizing conditions in LiCl (Fig. 2C, D). Consistent with the loss of G4 structure [47, 48], CD spectra for the corresponding mutated sequences of each G4 were markedly different compared to WT, with minima ~250 nm and maxima ~280 nm (Fig. 2C, D). Thermal difference spectra (TDS) [49] and UV thermal melting spectroscopy data for HS-40 and HS2 G4 oligonucleotides were also consistent with G4 structure formation [50] (Additional file 1: Fig. S1A, B). HS-40 and HS2 G4 oligonucleotides showed melting temperatures of 55.8 ± 0.1 °C and 44.4 ± 0.1 °C at 295 nm and 310 nm (respective differential maximum in TDS). No melting transition was observed for the corresponding mutant sequences (Additional file 1: Fig. S1B). Overall, these biophysical results confirm that the chosen mutations for the HS-40 and HS2 G4 s abrogate G4 folding in vitro.

We considered the possibility that our mutations to G4 sequences in the HS-40 and HS2 enhancers disrupted key protein–DNA interactions as a direct consequence of altering the primary sequence and GC-content of duplex DNA, as opposed to being G4-structure dependent. We investigated this possibility with respect to SP1, a key TF that binds GC-rich DNA and regulates globin gene expression in K562 cells [51, 52]. Notably, SP1 is also known to bind G4 structures with similar affinity to GC-rich duplex sequences [53]. We performed protein affinity capture from wild-type K562 cell nuclear lysates using WT G4 single-stranded DNA oligonucleotides alongside WT and mutated G4 duplex DNA oligonucleotides and assessed SP1 binding by western blotting. We found that SP1 did not bind the duplex form of either the WT or mutated G4 sequence motifs (Additional file 1: Fig. S2B). This suggests that the mutations introduced into these G4 motifs did not adversely affect SP1 binding to duplex DNA and thus the phenotypic effect of these mutations is unlikely to result from disruption of SP1 binding to duplex DNA at these sites.

We then set out to demonstrate that K562 cells carrying the equivalent mutations to those in vitro above would similarly attenuate G4 folding within the native genomic context of the α- and β-globin enhancer loci. Using CRISPR-Cas9 genome editing, we generated two cell lines homozygous for G4 mutations in HS-40, termed αMut1 and αMut2, and two cell lines homozygous for G4 mutations in HS2, termed βMut1 and βMut2. For each cell line clone, the desired mutations were confirmed by Sanger sequencing and potential off-target mutations ruled out using CRISPOR [54] (Additional file 1: Fig.S 2 A). We then evaluated G4 folding in αMut cells by G4 CUT&Tag with two independent G4-specific antibodies, BG4 and SG4 [11, 12], and observed a significant (*P*-value < 1E–07) reduction in G4 signal at HS-40 to 39% of wild-type levels (Fig. 2E, F). Similarly, for βMut cells, we also observed a reduction in folded G4 signal at HS2 to 30% of wild-type levels (*P*-value < 1E − 05) (Fig. 2G, H). The visible reduction in G4 signal in mutant cells was supported by peak calling using the SEACR algorithm (“stringent” 0.01) [55]. SEACR identified a G4 peak at either HS-40 or HS2 in almost all replicates in wild-type cells, but to a far lesser extent in the corresponding G4 mutant CUT&Tag libraries. Loss of G4 folding was confirmed in at least 2 independent clones for each cell line (Additional file 1: Fig. S3A). The residual levels of G4 signal in αMut and βMut cells may reflect known antibody-independent Tn5 activity in CUT&Tag [38]. Confirming the specificity of our G4 editing strategy, we found that the HS-40 and HS2 G4 CRISPR edits did not introduce aberrant editing at other G4s (e.g., KRAS, SDF4, and RPA3), that were not selected for genome editing (Fig. 2F–H and Additional file 1: Fig. S3B-D). Together these results confirm that the selected mutations in G4 encoding sequences markedly diminish endogenous G4 folding in the HS-40 and HS2 enhancers.

### Restoration of enhancer G4 structure formation by an unrelated G4 sequence

We next established whether a G4 encoded by a different primary sequence could impart G4 formation within an endogenous enhancer chromatin context. To exemplify this, we performed genome editing at the HS-40 α-globin enhancer in αMut1 cells to generate cells termed αRes that carry the well-characterized 27 bp G4 sequence from the *MYC* upstream regulatory element [5, 56] (Additional file 1: Fig. S4). By G4 CUT&Tag, it was readily apparent that G4 folding at HS-40 in αRes cells was largely restored to 81% of WT levels (Fig. 2E, F). Taken together, our results show that genome editing can remove folded G4 structures in HS-40 and HS2 enhancers, and G4 structure formation can be re-established via the introduction of a different G4 sequence.

### Folded G4s at enhancers promote chromatin accessibility

We next determined whether a folded G4 structure influences chromatin accessibility in the HS-40 and HS2 globin enhancers, as nucleosome organization is critical for enhancer activity [57–59] and it is known that G4s are involved in determining an open chromatin state in promoters [5, 60]. To evaluate this, we mapped chromatin accessibility by Cleavage Under Targeted Accessible Chromatin (CUTAC) [61] for open chromatin regions defined by H3K4me2 marked nucleosomes [62, 63] in WT and G4 mutated cells (Fig. 2I, J). In both αMut and βMut cells, the CUTAC signal at the HS-40 and HS2 enhancers was significantly reduced to ~35–37% of WT levels, respectively (Additional file 1: Fig. S3E,F; *P*-values < 0.05, < 0.01). This reduction was noted over the G4 motif itself and, in the case of HS-40, at the 3’ region of the enhancer that is 120 bp in length. Notably, in αRes cells, the CUTAC signal was restored to WT levels (Fig. 2I and Additional file 1: Fig. S3E). At control sites outside of the edited regions, we observed no difference in CUTAC signal (Additional file 1: Fig. S3G). Overall, these results show that the presence of a folded G4 structure contributes to chromatin accessibility at enhancers and suggests that G4s are part of a mechanism that maintains an active chromatin state to promote enhancer activity.

### G4s facilitate 3D α-globin enhancer-target gene interactions

**Fig. 3:**
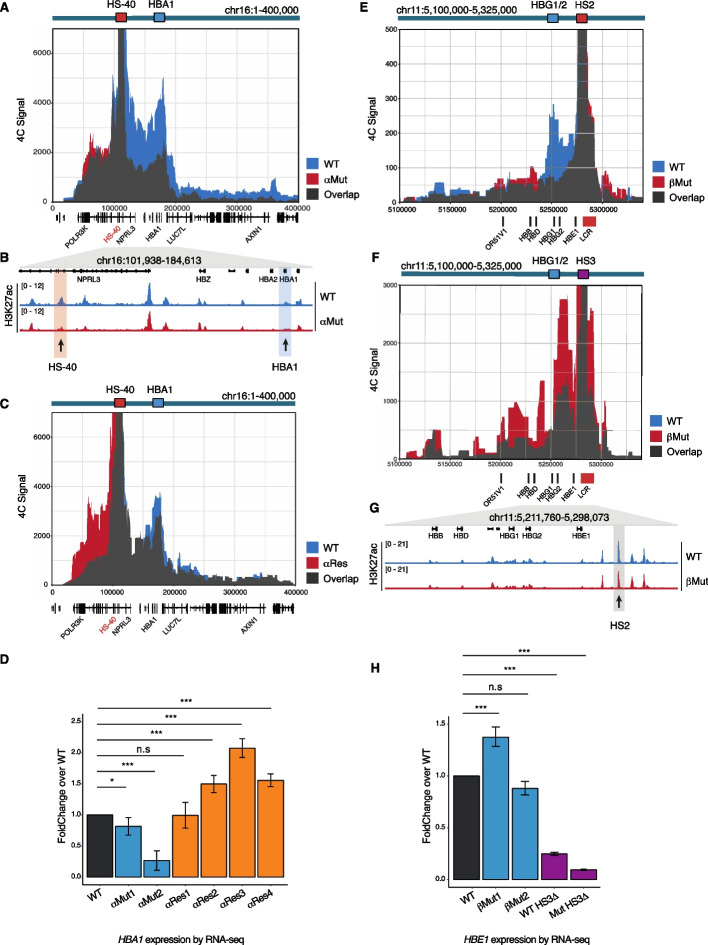
Loss of G4 structure in enhancers abrogates 3D chromatin interactions.** A** Chromatin conformation capture signal (4C-seq) from the viewpoint of the α-globin HS-40 enhancer in wild type (WT, blue) and G4 mutated (αMut, red) cells. Overlap is highlighted in gray. **B** IGV browser view of H3K27ac signal by CUT&Tag between the HS-40 enhancer and HBA1 target gene. Red shading indicates the HS-40 enhancer, blue shading indicates the *HBA1* gene. **C** 4C-seq signal from the viewpoint of the HS-40 enhancer in wild type (WT, blue) and G4 restored (αRes, red) cells. Overlap is indicated in gray. **D** Bar plot of differential expression analysis by RNA-seq for *HBA1* mRNA expression, fold change relative to wild type (WT, black) for G4 mutated cell lines (αMut1–2, blue), and G4 restored cell lines (αRes1–4, orange) is shown. Error bars represent the standard error (SE), *P-value < 0.05, ***P-value < 0.001, n.s is not significant. **E** 4C-seq signal from the viewpoint of the HS2 enhancer in wild type (WT, blue) and G4 mutated (βMut, red) cells. Overlap is indicated in gray. **F** 4C-seq signal from the viewpoint of the β-globin locus HS3 enhancer in wild type (WT, blue) and G4 mutated (βMut, red) cells. Overlap is indicated in gray. **G** IGV browser view of H3K27ac signal by CUT&Tag across the β-globin locus in wild type (WT) and G4 mutated (βMut) cells. Gray shading indicates the HS2 enhancer. **H** Bar plot of differential expression analysis by RNA-seq for *HBE1* mRNA expressed as fold change relative to wild type (WT, black) for G4 mutated cell lines (βMut1–2, blue), HS3 enhancer deleted (WT-HS3Δ, purple) and HS2 G4 mutated plus HS3 enhancer deleted cells (βMut-HS3Δ, purple). Error bars represent the standard error (SE), ***P-value < 0.001, n.s is not significant. See also Additional file 1: Figs. S5–S6

Whether G4 structures in globin enhancers directly influence enhancer activity remains an open question. To address this, we investigated how 3D contacts of the HS-40 α-globin enhancer and concomitant target gene expression were affected by G4 loss. Using Circular Chromosome Conformation Capture (4C-seq) [64], we demonstrated that in WT cells HS-40 3D contacts are distributed ~60 kb downstream and ~70 kb upstream (Fig. 3A). Upon loss of the HS-40 G4 in αMut cells, upstream contacts, including at the *HBA1* target gene, were reduced by ~50% whereas downstream contacts remained comparable to WT (Fig. 3A and Additional file 1: Fig. S5A). Reduced enhancer activity on G4 loss was also apparent by decreased deposition of the active enhancer histone mark H3K27ac at HS-40 as assessed by CUT&Tag (Fig. 3B). Strikingly, *HBA1* target gene expression was also reduced by ~40% on average across two biological clones (fold change over WT 0.27–0.81; *P*-value adj = 6.5E − 32, 0.04, respectively) (Fig. 3D). These findings suggest that loss of a folded G4 structure at HS-40 disrupts specific enhancer–promoter interactions resulting in dysregulated globin gene expression.

We then determined whether re-inserting a G4 of different primary sequence into G4 mutated cells restored 3D gene contacts and *HBA1* target gene expression. Using αRes cells above, which are edited to have the MYC G4 in HS-40, we found that 3D contacts with *HBA1* and target gene expression were restored (fold change over WT 0.99–2.07 across 4 biological clones; αRes1–4 *P*-value adj = 0.97, 2.19E − 04, 1.15E − 11, 1.72E − 05, respectively) (Fig. 3C, D, fold change for αMut1, 2 versus αRes cells in Additional file 1: Fig. S5B). Insertion of the MYC G4 resulted in elevated 3D contacts across the locus, which we reason is the result of insertion into the mutant HS-40 background. The observed clonal variability in gene expression here is comparable to prior work on the effect of globin enhancer deletions [32] and may have arisen from the selective pressures of single cell CRISPR clone generation; for completeness, we have shown all clones generated. Together, these findings show that a folded G4 structure in the HS-40 enhancer positively regulates α-globin gene expression via a mechanism that maintains key enhancer–promoter interactions.

### *The HS2 β-globin locus enhancer G4 co-operates with HS3 to promote target gene interactions*

We next considered the role of the G4 in the HS2 enhancer, which exhibits interdependency on other globin enhancers such as HS3 [34, 65, 66]. We first measured changes in 3D contacts at the HS2 β-globin locus enhancer upon HS2 G4 loss. Compared to WT cells, βMut cells displayed a ~80% reduction in 3D contacts between HS2 and the downstream target genes, including *HBE1* (Fig. 3E and Additional file 1: Fig. S5C). HS2 is critical for *HBE1* expression and, along with *HBG1/2*, is actively expressed in K562 cells, while *HBB* is not expressed. Due to the interplay between HS2 and HS3 enhancers, we next examined whether HS2 G4 loss also perturbs HS3 enhancer interactions. We measured specific contacts from HS3 to distal sites in 4C-seq and observed a marked increase in 3D contacts between HS3 and many downstream β-globin target genes in βMut cells (Fig. 3F and Additional file 1: Fig. S5D). However, no changes in the active enhancer histone mark H3K27ac across the LCR (Fig. 3G and Additional file 1: Fig. S5E) were observed. Furthermore, little change in *HBE1* target gene expression was seen on average (fold change over WT 0.88–1.38; *P*-value adj = 0.17, 5.47E − 05, respectively) (Fig. 3H).

These findings raise the question of whether the sustained expression of *HBE1* upon HS2 G4 loss could be mediated by HS3 hyperactivation. To address this, we generated further cell lines carrying a HS3 deletion alone (WT-HS3Δ) or both a HS3 deletion and HS2 G4 mutation (βMut-HS3Δ) (Additional file 1: Fig. S6A). When HS3 was deleted alone (WT-HS3Δ cells) *HBE1* expression was markedly reduced to ~25% (fold change over WT = 0.25, *P*-value adj = 1.90E − 130), whereas in cells without both HS2 G4 and HS3 (βMut-HS3Δ) *HBE1* expression was reduced further to only ~9% of WT levels (fold change over wild type 0.09; *P*-value adj = 2.34E − 143, fold change for βMut-HS3Δ versus WT-HS3Δ in Additional file 1: Fig. S6B) (Fig. 3H). This suggests that there is a combinatorial interplay between G4 structure in HS2 and the HS3 enhancer to maintain *HBE1* transcriptional output. This is also supported by the observed loss of enhancer-associated histone marks, H3K27ac and H3K4me1, at the HS2 enhancer observed in βMut-HS3Δ (~50% reductions compared to WT-HS3Δ cells *P*-value < 0.01–0.05) (Additional file 1: Fig. S6C, D). These results suggest that the HS2 enhancer maintains 3D interactions with distal regions through a G4 structure-based mechanism, but G4 structure loss in the HS2 enhancer leads to increased activity of HS3, possibly through epistatic mechanisms, to maintain target gene expression.

### Enhancer G4s bind and organize RNA polymerase II occupancy on chromatin

**Fig. 4:**
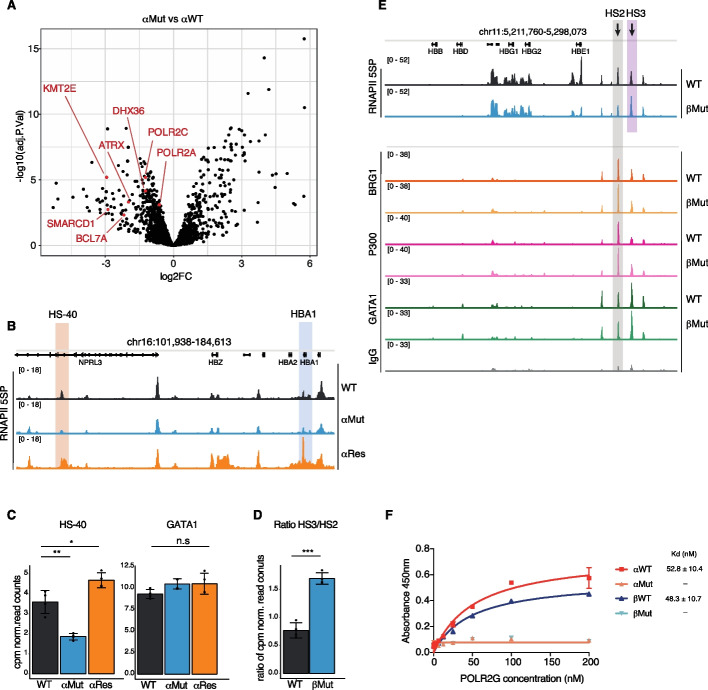
G4 structure binds RNAPII subunits and directs RNAPII occupancy on chromatin.** A** Volcano plot displaying proteins differentially enriched by affinity capture for α-globin HS-40 G4 mutated (αMut) vs. wild type (WT) folded G4 oligonucleotides. **B** IGV browser view of RNAPII 5SP signal by CUT&Tag across the α-globin locus; red shading indicates the HS-40 enhancer, and blue shading indicates the *HBA1* gene. **C** Bar plots of RNAPII 5SP cpm normalized read counts at the α-globin HS-40 enhancer and GATA1 promoter (*n* = 4) in wild type (WT), G4 mutated (αMut) and G4 restored (αRes) cells. Error bars represent the standard deviation (SD) *P-value < 0.05, **P-value < 0.01, n.s is not significant. **D** Bar plot of the ratio of cpm normalized read counts ratio HS3/HS2 for wild type (WT) vs. G4 mutated (βMut) cells (*n* = 4). Error bars represent the standard deviation (SD); ***P-value < 0.001. **E** IGV browser view for RNAPII 5SP, BRG1, P300, GATA1, and IgG signal by CUT&Tag across the β-globin locus; gray shading indicates the HS2 enhancer, and purple shading indicates the HS3 enhancer. **F** Binding curves determined by ELISA assay for the POLR2G subunit of the RNAPII complex to oligonucleotides for HS-40 (αWT) and HS2 (βWT) folded G4s and their corresponding mutant controls (αMut and βMut). Apparent K_d_ was calculated by one-site specific binding analysis. See also Additional file 1: S7

To gain further mechanistic insights into G4-mediated enhancer regulation, we investigated which proteins could interact with folded enhancer G4s. For this, biotinylated oligonucleotides for folded WT and mutated α-globin HS-40 G4s were used to affinity-capture interacting proteins from wild type K562 nuclear lysates followed by mass spectrometry analysis. Validating the method, known G4 binding proteins such as DHX36 [67] and ATRX [68] were found to be significantly enriched (*P*-value < 0.001) by αWT G4 compared to αMut oligonucleotides (Fig. 4A). In addition to G4 helicases, several validated G4-stabilizing proteins [69] were significantly enriched by the αWT G4 (Additional file 1: Fig. S7B).

In contrast, and of particular note, was the lack of significant enrichment (*p* > 0.05) of known 3D architectural proteins including YY1, CTCF, cohesin complex subunits SMC1A, SMC3, STAG2, or Mediator complex subunits MED1–24 (Additional file 1: Fig. S7A). This suggests that previously reported interactions between promoter G4s and these architectural proteins [14, 15] are not relevant for the globin enhancers that feature in our study. Supporting this contention, no signal for YY1 or CTCF at the HS-40 or HS2 enhancers is seen in ENCODE ChIP-seq data (Additional file 1: Fig. S7C,D).

HS-40 G4 oligonucleotides, compared to their mutated counterparts, significantly enriched several components of the RNA polymerase II (RNAPII) complex, such as POLR2A (*P*-value = 0.00079) and POLR2C (*P*-value = 6.5E − 06) (Fig. 4A). This also supports earlier findings of probable RNAPII-G4 interactions identified by global mass spectroscopy profiling of protein-DNA affinities [70] and the well-established role of RNAPII in enhancer RNA transcription [71, 72] and maintenance of 3D contacts [73, 74].

Given our results on G4s promoting enhancer function above together with the binding of RNAPII components by G4 oligonucleotides, we evaluated whether RNAPII recruitment to enhancers was dependent on the presence of a folded G4 structure. We therefore used CUT&Tag to map the initiating form of RNAPII (5SP) in WT and αMut cells at the HS-40 enhancer and found that RNAPII recruitment decreased by 50% in mutated relative to WT cells (*P*-value < 0.01) (Fig. 4B, C). In contrast, when we evaluated RNAPII 5SP levels in αRes cells that carry a MYC G4 in HS-40, we noted a marked increase of ~30% relative to WT cells (*P*-value < 0.05) (Fig. 4B, C). RNAPII 5SP distribution was also found to be reorganized in βMut cells (without the HS2 G4) as there was a relative increase in RNAPII 5SP recruitment at HS3 compared to WT (HS3/HS2 signal ratio increased from 0.7 in WT to 1.4 βMut cells, *P*-value < 0.0001) (Fig. 4D, E). This reorganization was specific to RNAPII 5SP as we observed no change in CUT&Tag signal of the lysine acetyltransferase P300 [75–77], the enhancer associated SWI/SNF ATPase subunit BRG1 (SMARCA4) [57, 59], or GATA1, a master regulator of erythroid gene regulation [78] at the β-globin LCR in βMut cells (Fig. 4E). This specific increase in RNAPII at HS3 is consistent with hyperactivation of HS3 observed by 4C-seq and gene expression (Fig. 3F, H). Overall, these results suggest that folded G4s facilitate RNAPII recruitment to key α- and β-globin locus enhancers to coordinate enhancer-mediated gene expression.

Given our findings that G4s are coupled with RNAPII recruitment at enhancers, we then set out to investigate whether a folded enhancer G4 directly interacts with RNAPII protein subunits. For this, we performed enzyme-linked immunosorbent assays (ELISAs) to determine any direct binding of full-length recombinant RNAPII components to immobilized G4 oligonucleotides or their mutated counterparts. We selected two subunits from diverse functional parts of the RNA polymerase II complex, POLR2C and POLR2G, which form part of the catalytic core and the mobile stalk, respectively [79, 80]. By ELISA, POLR2G showed selective binding to both WT α- and β-globin enhancer folded G4s compared to their mutated counterparts (α, apparent Kd 52.8 nM vs no detectable binding; β, apparent Kd = 48.3 nM vs no detectable binding Fig. 4F), supporting a direct G4 interaction. On the other hand, POLR2C showed no G4 binding (Additional file 1: Fig. S7E) suggesting an indirect association in our affinity enrichment experiments above (Fig. 4A). Overall, the direct binding of RNAPII subunit with G4s in vitro, and the G4-dependent recruitment of RNAPII to globin enhancers in cells, suggests that enhancer G4s promote gene expression through a mechanism whereby folded G4s enable RNAPII recruitment at enhancers.

## Discussion

Here, we report a functional role for G4 structures in α- and β-globin locus enhancer elements that facilitate their contact with target genes to promote globin gene expression. It is remarkable that small changes of a few base pairs at G4 forming sequences have profound effects on enhancer function comparable in magnitude to that of complete enhancer deletion [32]. By generating cells without an enhancer G4, or by replacing the endogenous G4 with a G4 of different primary sequence, we demonstrate that the ability to form G4 structure imparts positive regulatory activity to enhancers. Importantly, this discovery extends beyond the emerging role of G4 structure as positive regulators of transcription in gene promoters [5, 6, 81] as we now demonstrate that G4 structures located in globin enhancers also positively regulate gene expression by modulating chromatin accessibility, RNAPII recruitment, and 3D organization. Our study thus identifies a new class of chromatin features used by globin enhancers to interact with their target promoters.

Our analysis of enhancer-interacting proteins by CUT&Tag reveals that accurate RNAPII recruitment to the HS-40 and HS2 globin enhancers is linked to G4 structure formation. Moreover, our affinity enrichment and biophysical experiments with folded G4 oligonucleotides confirm that specific RNAPII complex subunits can interact directly with G4 structures. While our findings provide novel insights into the known roles of RNAPII in enhancer activity, these interactions may not be the only mechanism by which G4 structures positively influence enhancer function. As G4s are known to bind many chromatin factors, including nucleosome remodeller subunits [82] and transcription factors [16], G4s may also serve to recruit diverse protein effectors to enhancers.

The details of how a folded G4 structure facilitates enhancer–promoter interactions in 3D space and the precise order of molecular events remain to be determined. Here, we have taken the first steps to address these questions and have now established that G4 structure formation is positively linked to enhancer chromatin accessibility. This may represent a key regulatory mechanism by which G4s modulate enhancer chromatin to enable the subsequent recruitment of G4 interacting proteins such as RNAPII subunits. Likewise, how proteins that interact with G4s build 3D chromatin loops is unclear. Our affinity enrichment experiments with folded G4 oligonucleotides did not capture architectural proteins, such as YY1, CTCF, cohesin, or mediator subunits, which suggests that G4s in globin enhancers function through mechanisms independent of these factors to promote enhancer–promoter interactions.

## Conclusions

In conclusion, we have discovered that G4 structures are important structural features of α- and β-globin locus enhancers that mediate 3D enhancer–promoter interactions to support accurate gene expression. How general this mechanism applies to enhancers other than globin and in different cell types will be an important question for future studies. G4 enhancer-based mechanisms may be particularly relevant during the specification of cell type identity during development, as over 40% of folded G4 s are found in cell type-specific enhancers [3]. We thus suggest that G4 structures should now be considered in any model of enhancer function.

## Methods

### Cell culture

K562 cells (ATCC) were cultured in RPMI1640 (Glutamine plus, Life Technologies) with 10% of fetal bovine serum (Life Technologies) at 37 °C with 5% CO_2_. Cell cultures were routinely checked for mycoplasma infection. Cells were not further authenticated.

### Chromatin profiling by CUT&Tag

Bulk CUT&Tag was conducted as previously described [10, 38]. Dynabeads MyOne Streptavidin T1 beads (65,601, Thermo Fisher Scientific) were incubated with Concanavalin A (C2272, biotin conjugate, Sigma-Aldrich) in a ratio of 10 mg/mL beads to 1.15 mg/mL Concanavalin A at 22 °C for 30 min at 400 rpm on a thermoshaker (Eppendorf). Volume sufficient for 10 µL beads per reaction was prepared. Beads were washed with 1 mL of binding buffer (20 mM HEPES pH 7.5, 10 mM KCl, 1 mM CaCl_2_, 1 mM MnCl_2_) and resuspended in a volume of binding buffer sufficient for 10 mL beads per reaction. Cells were fixed in 0.1% formaldehyde (28,906, Thermo Fisher Scientific) in 1X PBS for 2 min at room temperature and quenched with 0.075 M glycine. Cells were centrifuged at 1300 × g 4 °C for 4 min and resuspended in wash buffer (20 mM HEPES pH 7.5, 150 mM KCl, 0.5 mM spermidine (S0266, Sigma-Aldrich), one tablet protease inhibitor (11,873,580,001, Roche cOmplete, EDTA-free)) at 500 cells/mL. Ten-milliliter beads were incubated with 100 mL of cell suspension at 25 °C for 10 min at 600 rpm, washed twice with 100 mL wash buffer and resuspended in 50 mL of antibody buffer (2 mM EDTA, 0.1% BSA (A8577, Sigma-Aldrich), 0.05% digitonin (300,410, Merck Millipore) in wash buffer). Primary antibody was added and incubated at 4 °C at 600 rpm overnight. Primary antibodies used include BG4 sc-Fv (made in house, as described previously [11]) at 0.5 mM per reaction, SG4 (made in house, as described previously [12]) at 1 mM per reaction, anti-H3K4me1 antibody (710,795, Thermo Fisher Scientific), anti-H3K27ac antibody (ab4729, Abcam), anti-Ser5 phosphorylated Rpb1 antibody (#13,523, Cell Signaling Technology), anti-GATA1 antibody (ab181544, Abcam), anti-BRG1 antibody (ab110641, Abcam), anti-P300 antibody (sc-48343, Santa Cruz Biotechnology), and Normal Rabbit IgG (2729, Cell Signaling Technology). Cells were washed twice with 100 mL dig wash buffer (0.05% digitonin in wash buffer) and resuspended in 50 mL of dig-wash buffer. Secondary antibody was then added and incubated at 25 °C at 600 rpm for 1 h. For BG4 and SG4 samples, 2 mL of rabbit anti-FLAG antibody (#2368, Cell Signaling Technology) was added; for all other rabbit primary antibodies, 0.5 mL of Guinea Pig anti-rabbit IgG antibody (ABIN101961, antibodies-online.com) was added, and for mouse primary antibodies, 0.5 mL of Rabbit Anti-mouse IgG H&L (ab46540, Abcam) was added per reaction. Cells were then washed three times with 500 mL dig-wash buffer and resuspended in 50 mL dig-300 buffer (20 mM HEPES pH 7.5, 300 mM KCl, 0.5 mM spermidine, 0.01% digitonin, one tablet protease inhibitor). pA-Tn5 (2 mM, made in house as described previously [10, 38]) was added in 1:250 dilution to each sample and incubated at 25 °C at 600 rpm for 1 h. Cells were washed three times with 500 mL dig-300 buffer and resuspended in 300 mL tagmentation buffer (10 mM MgCl_2_ in dig-300 buffer) and incubated at 37 °C at 600 rpm for 1 h. Cells were then washed twice with 500 mL TAPS wash buffer (10 mM TAPS (J63268.AE, Thermo Fisher Scientific) and resuspended in 100 mL of proteinase K solution (0.5 mg/mL Proteinase K (EO0491, Thermo Fisher Scientific), 0.5% SDS in 10 mM Tris–HCl pH 8.0). Samples were vortexed on full and incubated at 55 °C for 1 h at 800 rpm. DNA was extracted using DNA Clean & Concentrator 5 (D4013, Zymo Research) following the manufacturer’s instructions. DNA was eluted in 25 mL DNA elution buffer. Sequencing libraries were generated by mixing 25 mL NEBNext High Fidelity 2X PCR Master Mix (M0541, New England Biolabs), 2 mL of P5 indexing primer (10 mM), 2 mL of P7 indexing primer (10 mM) with 21 mL of purified DNA in a PCR reaction under the following conditions: 72 °C for 5 min, 98 °C for 30 s followed by n cycles of 98 °C for 10 s, 63 °C for 10 s, and a final extension at 72 °C for 1 min. For BG4 and SG4 libraries, *n* = 8 cycles were used; for all other CUT&Tag libraries, *n* = 10 was used. Libraries were purified using Ampure XP beads (A63882, Beckman Coulter Life Sciences) by incubation with 0.4 × ratio of beads for 15 min, followed by supernatant incubation with 1.4 × ratio of beads for 15 min. Beads were washed twice with 800 mL 80% ethanol and eluted in 25 mL of 10 mM Tris–HCl pH 8.0. Library size distribution was analyzed on a Tapestation 2200 (Agilent Technologies) and library concentrations were quantified by NEBNext Library Quant Kit for Illumina (E7630, New England BioLabs). Individual libraries were pooled for optimal read balancing and sequenced paired end with NextSeq 2000 P2 or P3 Reagents 100 Cycles v3 (20,046,811, 20,040,559, Illumina, Inc.) on the NextSeq 2000 platform (Illumina, Inc.).

### Chromatin accessibility mapping via CUTAC

Cleavage Under Targeted Accessible Chromatin (CUTAC) was performed as previously described [61] and following the CUT&Tag procedure outlined above with the following changes. The primary antibody used for all CUTAC samples was anti-dimethyl-Histone H3 (lys4) (07–030, Merck Millipore) 1:50 per reaction. pA-Tn5 catalyzed tagmentation was performed in 300 mL CUTAC buffer (10 mM TAPS (J63268.AE, Thermo Fisher Scientific) pH 8.5, 5 mM MgCl_2_) at 37 °C at 600 rpm for 20 min.

### CUT&Tag and CUTAC read mapping and processing

Libraries were demultiplexed using demuxFQ (flags: -c -d -i -e -t 1 -r 0.01 -R -l 9). FASTQC- version 0.11.8 was used to assess the read quality, and bases with a quality score below 20 were trimmed from both reads using cutadapt (cutadapt -q 20). For CUTAC libraries, reads greater than 120 bp in length were removed before alignment. Reads were aligned to the hg38 reference genome and corresponding mutant genomes using bwa mem with default parameters. Sorted bam files with duplicates removed were generated with SAMtools (version 1.15.1) and converted to bigwig using deepTools bamCoverage (parameters: –normalizeUsing CPM, –binSize 10, 5 or 3 with 12nt smoothing function). Statistical comparisons between WT and Mut and Res samples were performed by two-sided t-test using maximum cpm signal over 500 bp regions. Sample size for CUT&Tag samples is minimum of two replicates per condition; sample sizes for statistical analysis are indicated.

### 4C-seq library preparation and data analysis

4C-seq was performed by following the published protocol [64] with some modifications. Thirty million cells were crosslinked in 2% formaldehyde for 10 min at room temperature and quenched with 0.125 M glycine for 5 min. Cells were washed once in 50 mL of ice-cold PBS and centrifuged at 1500 rpm for 10 min at 4 °C. Nuclei were extracted by incubation in 50 mL cell lysis buffer (10 mM Tris–HCl pH 8, 10 mM NaCl, 0.2% IGEPAL CA-630 (18,896, Sigma-Aldrich), one tablet protease inhibitor (11,873,580,001, Roche cOmplete, EDTA-free) for 30 min on ice and centrifuged at 1800 rpm for 5 min at 4 °C. Nuclei were overlayed once with 500 mL of 1.25 × NEBuffer r3.1 (B6003S, New England Biolabs); buffer was removed, pellet was resuspended in 1 mL of 1.25 × NEBuffer r3.1, and aliquoted in 250 mL volumes. To each aliquot, 108 mL of 1.25 × NEBuffer r3.1 was added, followed by 11 mL of 10% SDS (V6551, Promega) and incubated at 37 °C for 1 h in a thermoshaker (Eppendorf) at 950 rpm. 75 mL of 10% Triton X-100 (93,443, Sigma-Aldrich) was added to each tube and incubated for 1 h at 37 °C at 950 rpm. Five hundred units of DpnII (R0543M, New England Biolabs) was added to each tube and incubated overnight at 37 °C. After centrifugation at 2500 rpm at 4 °C for 5 min, the pellet was resuspended in 950 mL of 1 × ligation buffer (15,224–025, Invitrogen), and 50 mL of 1U/mL T4 DNA ligase (15,224–025, Invitrogen) was added and incubated at 16 °C for 4 h, without shaking. Crosslinks were removed by the addition of 60 mL 10 mg/mL Proteinase K (03115879001, Roche) and incubated overnight at 65 °C with shaking at 800 rpm. Another 60 mL 10 mg/mL Proteinase K was added the following day and incubated at 65 °C at 800 rpm for 2 h. Ten milliliters of 10 mg/mL Rnase A (10,109,142,001, Roche) was then added, followed by incubation at 37 °C at 800 rpm for 1 h. Reaction volumes were pooled, and DNA was purified by phenol:chloroform extraction; equal volume of phenol pH 8.0 (P4557, Sigma-Aldrich) was added, vortexed for 1 min, and centrifuged at 3500 rpm for 10 min at room temperature. The aqueous phase was transferred to a new tube, and extraction was repeated as above with equal volume of phenol:chloroform pH 8.0 (P3803, Sigma-Aldrich). DNA was precipitated in 1/10 volume 3 M sodium acetate over 34 mL of ethanol overnight at − 20 °C. Precipitated DNA was centrifuged at 3900 rpm at 4 °C for 30 min and resuspended in 400 mL of 1 × TE buffer. DNA was purified again with an equal volume of phenol:chloroform as above; this was repeated twice in total, and DNA was precipitated in 0.1 × volume of 3 M sodium acetate over 1 mL of ethanol overnight at − 20 °C. Precipitated DNA was centrifuged at 14,000 rpm at 4 °C for 30 min, washed three times with 1 mL of 70% ethanol, and resuspended in 25 mL of 1 × TE buffer.

For the second restriction enzyme digestion, DNA was incubated with 500 U of DdeI (R0175L, New England Biolabs) in 1 × NEBuffer r3.1 in 500 mL total volume overnight at 37 °C at 500 rpm. Enzyme was heat inactivated by incubating for 20 min at 65 °C. Ligation reaction was performed in 1 × ligation buffer and 50 U 1U/mL T4 DNA ligase in total volume of 5 mL overnight at 16 °C, without shaking. DNA was purified by phenol:chloroform following steps outlined above. First round PCR for specific enhancer sites was performed in four (for HS2 and HS3 viewpoints) or ten (for HS-40 viewpoint) parallel reactions per enhancer site using 25 mL NEBNext High-Fidelity 2X PCR Master Mix (M0541 New England Biolabs), 2 mL 10 mM 4 C forward primer, 2 mL 10 mM 4 C reverse primer, and 200 ng of 4 C template in total 50 mL volume, under the following conditions: 72 °C for 3 min, 98 °C for 1 min followed by 16 cycles of 98 °C for 10 s, primer specific Ta for 30 s, 72 °C for 1 min, and final extension at 72 °C for 2 min. Four reactions were pooled and purified with 1.3X Ampure XP Beads (A63882, Beckman Coulter Life Sciences) and eluted in 100 mL 10 mM Tris–HCl pH 8.0. For addition of Illumina adaptor sequences and dual indexes to amplified 4 C template, DNA from round 1 PCR was divided in four 21 mL volumes to which 25 mL NEBNext High-Fidelity 2X PCR Master Mix, 2 mL 10 mM forward adaptor primer, 2 mL 10 mM reverse adaptor primer was added to each reaction and amplified under conditions: 98 °C for 30 s, followed by 12 cycles of 98 °C for 10 s, 63 °C for 10 s, 72 °C for 30 s, and final extension at 72 °C for 2 min. Reactions were pooled and purified with 1.3 × Ampure XP Beads. Library size distribution was analyzed on a Tapestation 2200 (Agilent Technologies) and library concentrations were quantified by NEBNext Library Quant Kit for Illumina (E7630, New England BioLabs). 4 C libraries were multiplexed for next-generation sequencing on the NextSeq 2000 platform (paired-end: 76 bp and 36 bp reads). Biological replicates were performed together and sequenced together in a single run.

4C-seq data was analyzed using the pipe4 C pipeline (version 1.1.6) (https://github.com/deLaatLab/pipe4C) in R (version 4.1.0) Read 1 sequencing reads were used as input for the pipe4 C pipeline, and all reads mapped to the viewpoint chromosome were scaled to 1 million reads. An average running mean window size was set at 21 fragment ends. 4 C plots were generated using peakC [83] (https://github.com/deWitLab/peakC) where two biological replicates were used as input into the peakC program and subsequently plotted with ggplot2 in R. Biological replicates were processed on the same day and sequenced on the same flow cell. Samples for comparison were sequenced together in a single run.

### Oligonucleotide biophysical characterization

Oligonucleotides were diluted in either potassium (10 nM lithium cacodylate, 100 mM KCl) or lithium (10 nM lithium cacodylate, 100 mM LiCl) buffer, heated (95 °C, 5 min) and cooled overnight. For circular dichroism spectroscopy, 150 mL oligonucleotide solution was analyzed in triplicate on the Chirascan VX Circular Dichroism Spectrometer (Applied Photophysics) (24 °C, 220–320 nm, 0.3 nm steps, 1 mm path length). The spectrum was smoothened (Chirascan software, 10 measurement window) and background signal was removed. For UV spectroscopy and melting, 500 mL oligonucleotide solution per sample was loaded into a 1 cm path length UV cuvette and topped with 200 mL mineral oil. UV measurements were collected using the Cary 3500 Peltier UV–Vis Spectrophotometer (Agilent)—spectra were first collected at 25 °C and 95 °C, followed by thermal melting (25–95 °C, 0.5 °C per minute, 0.2 °C measurement steps, absorbance taken at 245 nm, 295 nm 450 nm). For melting temperature calculation, data were analyzed using the Boltzmann sigmoidal as a model and plotted (GraphPad Prism 7). For thermal difference spectra, UV spectra collected at 95 °C and 15 °C were corrected using blank subtraction and both spectra were subtracted, and the resulting spectra were plotted (GraphPad Prism 7).

### G4 oligonucleotide affinity capture of protein interactors with Simple Western and LC–MS/MS analysis

Biotinylated oligonucleotides were folded by heating for 5 min at 95 °C and cooled gradually to 25 °C. Nuclear lysates were prepared with 90 million K562 cells per replicate. Cells were centrifuged (1000 × g, 4 °C, 5 min) and resuspended in 2.7 mL low salt buffer (20 mM HEPES–KOH pH 7.4, 10 mM NaCl, 3 mM MgCl_2_, 0.2 mM EDTA, 1 mM DTT in nuclease-free water with Roche Complete inhibitor tablets (11,873,580,001, Roche cOmplete, EDTA-free)), and incubated for 15 min on ice. One hundred thirty-five milliliters of 10% IGEPAL CA-630 (18,896, Sigma-Aldrich) were added in total and vortexed for 1 min. The cell extract was centrifuged (900 × g, 15 min, 4 °C) and the resulting nuclear pellet was washed with low salt buffer. The extract was centrifuged again, and nuclei were washed in 750 mL high salt buffer (20 mM HEPES–KOH pH 7.4, 500 mM NaCl, 3 mM MgCl_2_, 0.2 mM EDTA, 0.5% IGEPAL CA-630, 1 mM DTT in nuclease-free water with Roche Complete inhibitor tablets). The suspension was incubated on ice for 15 min with intermittent vortexing, followed by sonication to break up genomic DNA (10 cycles, 30 s on, 30 s off, at 4 °C, high amplitude on the Bioruptor Plus sonication device (Diagenode). The resulting lysate was centrifuged (16,000 × g, 10 min, 4 °C) and quantified using the Direct Detect Spectrometer (Merck Millipore). For lysate preclearing, 50 mL Magnesphere Streptavidin (Z5481, Promega) beads per sample were washed five times with 1 mL pulldown buffer (25 mM HEPES–KOH pH 7.4, 10.5 mM NaCl, 110 mM KCl, 1 mM MgCl_2_, 20% glycerol. 0.01 mM ZnCl_2_, 0.1% IGEPAL CA-630, 3% BSA, 1 mM DTT in nuclease-free water with Roche Complete inhibitor tablets). Lysate containing 250 mg protein was added to 50 mL beads and incubated for 2 h at 4 °C with rotation. Folded oligonucleotides were immobilized by addition of 50 mL of 10 mM oligonucleotide solution to 500 mL of pulldown buffer and incubated with 50 mL Magnesphere Streptavidin beads (pre-washed twice with 1 mL pulldown buffer) at RT for 30 min with rotation. This oligonucleotide immobilization step was omitted for bead only controls. The beads were then washed thrice with 2 mL of pulldown buffer and resuspended in 50 mL of pulldown buffer. Next, 500 mL of the precleared lysate was mixed with 10 mL 10 mg/mL salmon sperm DNA (15,632,011, Thermo Fisher Scientific) and added to 50 mL immobilized oligonucleotide-bead sample and incubated overnight at 4 °C with rotation. The next day, beads were washed six times in 1 mL pulldown buffer, followed by two washes in 1 mL 100 mM NH_4_HCO_3_ before probing antibody probing on the Simple Western platform (Protein Simple) or submission for label-free LC–MS/MS analysis, *n* = 4 for each pulldown sample.

For antibody detection on the Simple Western Wes platform (ProteinSimple), magnetic beads were resuspended in 25 mL of 1X LDS buffer containing 50 mM DTT and heated at 70 °C for 10 min to elute proteins, and beads were removed on magnetic. 1 mL of elution was analyzed on the Simple Western Wes platform (ProteinSimple) following the manufacturer’s instructions using anti-SP1 antibody (21,962–1-AP, Proteintech) in a 1:50 dilution. Protein detection was analyzed using the Compass for SW software package (ProteinSimple).

For label-free LC–MS/MS analysis, beads were digested with trypsin (Pierce) and the peptides were purified with Ultra-Micro C18 Spin Columns (Harvard Apparatus) prior to mass spectrometry analysis, as previously described (https://www.nature.com/articles/s41467-018-04619-5). Dried peptides were reconstituted in 0.1% formic acid and analyzed with an UltiMate 3000 UHPLC system coupled with the Q-Exactive HF (Thermo Scientific) mass spectrometer. The full scans were performed in the Orbitrap in the range of 400 to 1600 m/z at 60 k resolution. For MS2 scans, a resolution of 30 k was selected with a 2.0 Th isolation window and HCD collision energy of 28%. MS2 spectra were searched against a human UniProt database containing over 20,000 entries in Proteome Discoverer 2.4. The SequestHT node included the following parameters: Precursor Mass Tolerance of 20 ppm, Fragment Mass Tolerance of 0.02 Da, and Dynamic Modifications were Oxidation of M (+ 15.995 Da) and Deamidation of N/Q (+ 0.984 Da). The confidence level for peptide identifications was estimated with the Percolator node using target-decoy database search. Label-free quantification was performed with the Minora Feature Detector node. The consensus workflow included the Feature mapper and the Precursor Ion Quantifier node using intensity for precursor quantification. Only unique peptides were used for downstream statistical analysis. Label-free quantification was used. First, data was normalized using within-group median scaling, using the bead-only control as one group and all other pulldowns combined as the other (this included all pulldown conditions that were tested in this project). Only peptides found in over half of all pulldown replicates were analyzed further. Missing values were imputed in two ways—based on the minimum value in each sample (for all the bead-only controls and in pulldown samples for peptides found in less than 3 replicates) or using a *k*-nearest neighbors algorithm (in pulldown samples for peptides found in 3 or more replicates). A Limma-based differential abundance analysis was used to only select proteins enriched in any of the pulldown sets compared to the bead-only control for further analysis. Sample similarity was analyzed using hierarchical clustering analysis and principal component analysis. A replicate batch effect was seen affecting the dataset based on clustering and PCA analysis. Differentially abundant proteins between conditions were found using Limma-based analysis, with a significance threshold of *p* < 0.05, with replicates factored in for batch correction. HS-40 and HS2 G4 s are both parallel 3-tetrad G4 structures. We reasoned that in vitro affinity capture experiments with purified oligonucleotides that both G4 s would most likely bind a set of similar interacting proteins. We therefore decided to focus on the robust identification of interacting proteins in an extensive series of well-controlled mass spectroscopy experiments for the HS-40 G4 and its mutant counterpart in potassium buffers.

### Oligonucleotide enzyme-linked immunosorbent assay

Biotinylated oligonucleotides were folded in Tris–HCl pH 7.4, 100 mM KCl by heating for 5 min at 95 °C and cooled gradually to 25 °C. 50 nM of oligonucleotides were incubated on a streptavidin-coated 96-well plate for 1 h at room temperature. The plate was then blocked in 3% w/v BSA in ELISA buffer (100 mM KCL, 50 mM KH_2_PO_4_ adjusted to pH 7.4 with KOH) for 1 h at room temperature. Two hundred nanomolars of full-length recombinant human GST-tagged POLR2G protein (H00005436, Novus Biologicals) or full-length recombinant human His-tagged POLR2 C (NBP2-51,736, Novus Biologicals) was added to the first well, serially diluted in 50 mL volumes, and then incubated for 1 h at 450 rpm. The plate was washed three times in ELISA buffer plus 0.1% Tween20, and then incubated with anti-GST HRP-conjugated antibody (ab3416, Abcam) or anti-His HRP-conjugated antibody (BioLegend, 652,503) diluted 1:5000 in 3% w/v BSA in ELISA buffer. 3,3′,5,5′-tetramethylbenzidine ELISA substrate (slow kinetic rate) (Abcam, ab171525) was then added in 100 mL volume per well and incubated for 2 min, followed by the addition of 50 mL of 2 M HCl and color allowed to develop for 2 min. The plate was then analyzed on a PHERAstar plate reader at 450 nm absorbance. The equilibrium dissociation constant (Kd) values were calculated assuming a one-site binding model, *Y* = Bmax*X/(Kd + X), in GraphPad Prism, and the standard error of means of two replicates are shown.

### CRISPR genome editing for G4 substitution mutations and enhancer deletion

For cloning of CRISPR vectors for G4 substitution mutation and re-insertion, 2–3 guides per targeted locus were selected using CRISPOR, where guides had a high specificity score > 80 and the least number of predicted off-targets. Guides were cloned into PX458 (48,138, Addgene, a gift from Feng Zhang) SpCas9-2 A-EGFP and single guide RNA. For HS3 deletion, 4 guides were cloned into PX462 (62,987, Addgene, a gift from Feng Zhang) pCas9n-2 A-Puro (D10 A nickase) and single guide RNA. Briefly, oligos were phosphorylated with T4 polynucleotide kinase (M0201S, New England Biolabs) and annealed, while the plasmid backbone was digested with *Bbs*I and purified with Ampure XP beads (A63882, Beckman Coulter Life Sciences). 50 ng of the digested plasmid was ligated with the annealed insert using Quick Ligase (M2200S, New England Biolabs). The plasmid was then transformed into DH5a Competent *E. coli* (C2978H, New England Biolabs) following the manufacturer’s instructions, and the plasmid was extracted using the Monarch Plasmid MiniPrep Kit (T1010S, New England Biolabs) as per the manufacturer’s instructions and confirmed as containing the insert using Sanger sequencing.

For plasmid transfection, 500,000 K562 cells were seeded in 2 mL media per well in a 6-well plate. The following day, cells in each well were transfected with 2 mg of the relevant sgRNA-containing plasmid, together with 4 mL 10 mM 200 bp Ultramer (Integrated DNA Technologies) knock-in templates with homologous flanks, using Lipofectamine LTX Reagent with PLUS Reagent (A12621, Invitrogen, used as per manufacturer instructions for 500,000 suspension cells).

For cell sorting 48 h post transfection, cells were centrifuged (280 × g, 3 min) and washed in 1X PBS containing 1% FBS (A5209502, Thermo Fisher Scientific). Cells were resuspended in 2 mL PBS plus 1% FBS, strained, and sorted into 96-well plates with one cell per well using the BD FACSMelody Cell Sorter (BD Biosciences), selecting for cells with a GFP signal that was an order of magnitude more intense than the autofluorescence observed for mock transfected K562 control cells. Approximately 1.5–3% of cells sorted passed this criterion; ten 96-well plates, with 200 μL media in each well, were seeded per locus.

For clone selection, wells were visually inspected for clone survival after 14 days and passaged into another 96-well plate. Two or three plates were obtained per locus. Three days later, this plate was re-plated, and one plate lysed for genotyping as follows. Plates were centrifuged (1000 × g, 5 min, 4 °C) and media were removed. Twenty microliters of QuickExtract DNA Extraction Solution 1.0 (SS000035-D2, Lucigen) was added, and the cells were lysed (65 °C for 15 min, 68 °C for 15 min, 98 °C for 10 min) in PCR plates. 2.5 mL of the lysate per well was added to 25 mL OneTaq 2X master mix (M0482L, New England Biolabs) and 1 mL 10 mM of the forward and reverse genotyping primer, topped up with water to a 50 mL reaction in another 96-well plate. Primers were designed using the NCBI Primer-BLAST tool, aiming for a fragment size of 300–500 bp with minimal off-target amplification. At least two primer sets were tested per locus. The edited locus was amplified using PCR as per the OneTaq standard protocol, with an annealing temperature of 60 °C. Twenty-five microliters of the PCR reaction was digested with the relevant restriction enzyme at a concentration of 10 U per reaction with the relevant buffer (rCutSmart, NEB, B6004S) in a 40 mL reaction for 6 h, followed by heat deactivation of the enzyme. The digested product was analyzed on a 2% agarose gel containing SYBR Safe DNA stain (S33102, Thermo Fisher Scientific) in 0.5 × TBE buffer and imaged using the Amersham Imager 680 (GE Life Sciences). Clones with digestion patterns expected of the insert were submitted for PCR product sequencing (Source Bioscience) following PCR product cleanup using the QIAquick PCR Purification Kit (28,104 Qiagen), as per manufacturer instructions).

### RNA-seq and differential expression analysis

RNA from 1 × 10^6^ cells was extracted with RNeasy Plus Mini Kit (74,136, Qiagen) following the manufacturer’s instructions. 500 ng RNA was used as input for library preparation with NEBNext Ultra II Directional RNA Library Prep Kit for Illumina (E7760S, New England Biolabs), following the manufacturer’s instructions. Polyadenylated transcripts were enriched using the NEBNext Poly(A) mRNA Magnetic Isolation Module (E7490, New England Biolabs) as per the manufacturer’s protocol. Libraries were sequenced in paired-end mode on a NextSeq 2000 (51 bp paired-end). Paired-end files were demultiplexed using demuxFQ (flags: -c -d -i -e -t 1 -r 0.01 -R -l 9). FASTQC version 0.11.8 was used to assess the read quality and bases with a quality score below 20 were trimmed from both reads using cutadapt (cutadapt -q 20). Genomic annotations (gtf file) were downloaded from Gencode project portal (https://ftp.ebi.ac.uk/pub/databases/gencode/Gencode_human/release_42). Filtered paired-end reads were then aligned to the hg38 reference genome using bwa mem with default parameters. Sorted bam files were generated with SAMtools (version 1.15.1). Deduplication of the resulting mapped reads was performed with Picard MarkDuplicates (v2.18.7) (http://broadinstitute.github.io/picard/). Read quantification was done using the featureCounts function from the Subread package. Differential gene expression analysis was performed with the DESeq2 package in R (version 1.38.0) 92 on the raw read counts. Genes with an average of fewer than 10 reads per sample were omitted from downstream analysis. Benjamini & Hochberg correction was performed with significance thresholds |log2 FoldChange|> 0.5 and adjusted *P*-value (Padj) < 0.05. Sample size for RNA-seq: *n* = 8 for cell lines; αWT, αMut1, αMut2, αRes1, αRes2 and *n* = 4 for cell lines; αRes3, αRes4, βWT, βMut1, βMut2, WT HS3Δ, and Mut HS3Δ.

## Supplementary Information

Additional file 1: Fig. S1-S7. Supplementary figures.

Additional file 2: Table S1. Excel table of all results of differential analysis of protein affinity capture experiments.

Additional file 3: Table S2. Excel table of oligonucleotide sequences used in the paper.

## Data Availability

Genomics data is deposited in NCBI Gene Expression Omnibus under accession code: GSE26766 [84]. The mass spectrometry proteomics data have been deposited to the ProteomeXchange Consortium via the PRIDE [85] partner repository with the dataset identifier PXD063882. Results of differential analysis of protein affinity capture experiments is provided in Additional file 2: Table S1. All oligonucleotide sequences used in this study are provided in Additional file 3: Table S2. The following ENCODE datasets were used: ENCSR858MPS (K562 STARR-seq) [37, 86] ENCSR000EGM (file: ENCFF322EGW K562 CTCF ChIP-seq) [87], ENCSR000EWF (file: ENCFF577NSA, K562 YY1 ChIP-seq) [88]. Code for CUT&Tag, RNA-seq and 4C-seq analysis are available on GitHub [89] and Zenodo [90] under a Creative Commons Zero v1.0 Universal License.

## References

[1] Furlong EEM, Levine M. Developmental enhancers and chromosome topology. Science. 2018;361:1341–5. 30262496 10.1126/science.aau0320 PMC6986801

[2] Schoenfelder S, Fraser P. Long-range enhancer–promoter contacts in gene expression control. Nat Rev Genet. 2019;20:437–55. 31086298 10.1038/s41576-019-0128-0

[3] Zyner KG, Simeone A, Flynn SM, Doyle C, Marsico G, Adhikari S, Portella G, Tannahill D, Balasubramanian S. G-quadruplex DNA structures in human stem cells and differentiation. Nat Commun. 2022;13:142. 35013231 10.1038/s41467-021-27719-1 PMC8748810

[4] Lyu J, Shao R, Kwong Yung PY, Elsässer SJ. Genome-wide mapping of G-quadruplex structures with CUT&Tag. Nucleic Acids Res. 2022;50: e13. 34792172 10.1093/nar/gkab1073 PMC8860588

[5] Esain-Garcia I, Kirchner A, Melidis L, Tavares RDCA, Dhir S, Simeone A, Yu Z, Madden SK, Hermann R, Tannahill D, Balasubramanian S. G-quadruplex DNA structure is a positive regulator of MYC transcription. Proc Natl Acad Sci. 2024;121:e2320240121. 38315865 10.1073/pnas.2320240121 PMC10873556

[6] Chen Y, Simeone A, Melidis L, Cuesta SM, Tannahill D, Balasubramanian S. An upstream G-quadruplex DNA structure can stimulate gene transcription. ACS Chem Biol. 2024;19:736–42. 38417105 10.1021/acschembio.3c00775 PMC10949190

[7] Spiegel J, Adhikari S, Balasubramanian S. The structure and function of DNA G-quadruplexes. Trends Chem. 2020;2:123–36. 32923997 10.1016/j.trechm.2019.07.002 PMC7472594

[8] Hänsel-Hertsch R, Beraldi D, Lensing SV, Marsico G, Zyner K, Parry A, Di Antonio M, Pike J, Kimura H, Narita M, et al. G-quadruplex structures mark human regulatory chromatin. Nat Genet. 2016;48:1267–72. 27618450 10.1038/ng.3662

[9] Hänsel-Hertsch R, Spiegel J, Marsico G, Tannahill D, Balasubramanian S. Genome-wide mapping of endogenous G-quadruplex DNA structures by chromatin immunoprecipitation and high-throughput sequencing. Nat Protoc. 2018;13:551–64. 29470465 10.1038/nprot.2017.150

[10] Hui WWI, Simeone A, Zyner KG, Tannahill D, Balasubramanian S. Single-cell mapping of DNA G-quadruplex structures in human cancer cells. Sci Rep. 2021;11:23641. 34880271 10.1038/s41598-021-02943-3 PMC8654944

[11] Biffi G, Tannahill D, McCafferty J, Balasubramanian S. Quantitative visualization of DNA G-quadruplex structures in human cells. Nat Chem. 2013;5:182–6. 23422559 10.1038/nchem.1548 PMC3622242

[12] Galli S, Melidis L, Flynn SM, Varshney D, Simeone A, Spiegel J, Madden SK, Tannahill D, Balasubramanian S. DNA G-quadruplex recognition in vitro and in live cells by a structure-specific nanobody. J Am Chem Soc. 2022;144:23096–103. 36488193 10.1021/jacs.2c10656 PMC9782783

[13] Yu Z, Spiegel J, Melidis L, Hui WWI, Zhang X, Radzevičius A, Balasubramanian S. Chem-map profiles drug binding to chromatin in cells. Nat Biotechnol. 2023;41(9):1265–71. 36690761 10.1038/s41587-022-01636-0 PMC10497411

[14] Li L, Williams P, Ren W, Wang MY, Gao Z, Miao W, Huang M, Song J, Wang Y. YY1 interacts with guanine quadruplexes to regulate DNA looping and gene expression. Nat Chem Biol. 2021;17:161–8. 33199912 10.1038/s41589-020-00695-1 PMC7854983

[15] Wulfridge P, Yan Q, Rell N, Doherty J, Jacobson S, Offley S, Deliard S, Feng K, Phillips-Cremins JE, Gardini A, Sarma K. G-quadruplexes associated with R-loops promote CTCF binding. Mol Cell. 2023;83:3064-3079.e3065. 37552993 10.1016/j.molcel.2023.07.009 PMC10529333

[16] Spiegel J, Cuesta SM, Adhikari S, Hänsel-Hertsch R, Tannahill D, Balasubramanian S. G-quadruplexes are transcription factor binding hubs in human chromatin. Genome Biol. 2021;22:117. 33892767 10.1186/s13059-021-02324-z PMC8063395

[17] Kassouf M, Ford S, Blayney J, Higgs D. Understanding fundamental principles of enhancer biology at a model locus. BioEssays. 2023;45(10):2300047. 37404089 10.1002/bies.202300047 PMC11414744

[18] Bartman CR, Hsu SC, Hsiung CC, Raj A, Blobel GA. Enhancer regulation of transcriptional bursting parameters revealed by forced chromatin looping. Mol Cell. 2016;62:237–47. 27067601 10.1016/j.molcel.2016.03.007 PMC4842148

[19] Deng W, Lee J, Wang H, Miller J, Reik A, Gregory PD, Dean A, Blobel GA. Controlling long-range genomic interactions at a native locus by targeted tethering of a looping factor. Cell. 2012;149:1233–44. 22682246 10.1016/j.cell.2012.03.051 PMC3372860

[20] Deng W, Rupon JW, Krivega I, Breda L, Motta I, Jahn KS, Reik A, Gregory PD, Rivella S, Dean A, Blobel GA. Reactivation of developmentally silenced globin genes by forced chromatin looping. Cell. 2014;158:849–60. 25126789 10.1016/j.cell.2014.05.050 PMC4134511

[21] Williams TN, Weatherall DJ. World distribution, population genetics, and health burden of the hemoglobinopathies. Cold Spring Harb Perspect Med. 2012;2: a011692. 22951448 10.1101/cshperspect.a011692 PMC3426822

[22] Higgs DR, Wood WG, Jarman AP, Sharpe J, Lida J, Pretorius IM, Ayyub H. A major positive regulatory region located far upstream of the human alpha-globin gene locus. Genes Dev. 1990;4:1588–601. 2253879 10.1101/gad.4.9.1588

[23] Grosveld F, Van Assendelft GB, Greaves DR, Kollias G. Position-independent, high-level expression of the human β-globin gene in transgenic mice. Cell. 1987;51:975–85. 3690667 10.1016/0092-8674(87)90584-8

[24] Vernimmen D. Uncovering enhancer functions using the α-Globin locus. PLoS Genet. 2014;10: e1004668. 25330308 10.1371/journal.pgen.1004668 PMC4199490

[25] Fraser P, Hurst J, Collis P, Grosveld F. DNaseI hypersensitive sites 1, 2 and 3 of the human beta-globin dominant control region direct position-independent expression. Nucleic Acids Res. 1990;18:3503–8. 2362805 10.1093/nar/18.12.3503 PMC331003

[26] Palstra R-J, Tolhuis B, Splinter E, Nijmeijer R, Grosveld F, De Laat W. The β-globin nuclear compartment in development and erythroid differentiation. Nat Genet. 2003;35:190–4. 14517543 10.1038/ng1244

[27] Carter D, Chakalova L, Osborne CS, Dai Y-F, Fraser P. Long-range chromatin regulatory interactions in vivo. Nat Genet. 2002;32:623–6. 12426570 10.1038/ng1051

[28] Tolhuis B, Palstra R-J, Splinter E, Grosveld F, De Laat W. Looping and interaction between hypersensitive sites in the active β-globin locus. Mol Cell. 2002;10:1453–65. 12504019 10.1016/s1097-2765(02)00781-5

[29] Oudelaar AM, Davies JOJ, Hanssen LLP, Telenius JM, Schwessinger R, Liu Y, Brown JM, Downes DJ, Chiariello AM, Bianco S, et al. Single-allele chromatin interactions identify regulatory hubs in dynamic compartmentalized domains. Nat Genet. 2018;50:1744–51. 30374068 10.1038/s41588-018-0253-2 PMC6265079

[30] Oudelaar AM, Beagrie RA, Gosden M, De Ornellas S, Georgiades E, Kerry J, Hidalgo D, Carrelha J, Shivalingam A, El-Sagheer AH, et al. Dynamics of the 4D genome during in vivo lineage specification and differentiation. Nat Commun. 2020;11:2722. 32483172 10.1038/s41467-020-16598-7 PMC7264236

[31] Hay D, Hughes JR, Babbs C, Davies JOJ, Graham BJ, Hanssen LLP, Kassouf MT, Oudelaar AM, Sharpe JA, Suciu MC, et al. Genetic dissection of the α-globin super-enhancer in vivo. Nat Genet. 2016;48:895–903. 27376235 10.1038/ng.3605 PMC5058437

[32] Mettananda S, Fisher CA, Hay D, Badat M, Quek L, Clark K, Hublitz P, Downes D, Kerry J, Gosden M, et al. Editing an α-globin enhancer in primary human hematopoietic stem cells as a treatment for β-thalassemia. Nat Commun. 2017;8:424. 28871148 10.1038/s41467-017-00479-7 PMC5583283

[33] Fang X, Yin W, Xiang P, Han H, Stamatoyannopoulos G, Li Q. The higher structure of chromatin in the LCR of the β-globin locus changes during development. J Mol Biol. 2009;394:197–208. 19781549 10.1016/j.jmb.2009.09.046 PMC2849742

[34] Fang X, Xiang P, Yin W, Stamatoyannopoulos G, Li Q. Cooperativeness of the higher chromatin structure of the β-globin locus revealed by the deletion mutations of DNase I hypersensitive site 3 of the LCR. J Mol Biol. 2007;365:31–7. 17056066 10.1016/j.jmb.2006.09.072 PMC2826273

[35] Fang X, Sun J, Xiang P, Yu M, Navas PA, Peterson KR, Stamatoyannopoulos G, Li Q. Synergistic and additive properties of the beta-globin Locus Control Region (LCR) revealed by 5′HS3 deletion mutations: implication for LCR Chromatin Architecture. Mol Cell Biol. 2005;25:7033–41. 16055715 10.1128/MCB.25.16.7033-7041.2005 PMC1190234

[36] Bungert JR, Tanimoto K, Patel S, Liu Q, Fear M, Engel JD. Hypersensitive site 2 specifies a unique function within the human β-globin locus control region to stimulate globin gene transcription. Mol Cell Biol. 1999;19:3062–72. 10082573 10.1128/mcb.19.4.3062 PMC84100

[37] ENCODE Project Consortium. An integrated encyclopedia of DNA elements in the human genome. Nature. 2012;489:57–74. 10.1038/nature11247 PMC3439153 22955616

[38] Kaya-Okur HS, Janssens DH, Henikoff JG, Ahmad K, Henikoff S. Efficient low-cost chromatin profiling with CUT&Tag. Nat Protoc. 2020;15:3264–83. 32913232 10.1038/s41596-020-0373-x PMC8318778

[39] Arnold CD, Gerlach D, Stelzer C, Boryń ŁM, Rath M, Stark A. Genome-wide quantitative enhancer activity maps identified by STARR-seq. Science. 2013;339:1074–7. 23328393 10.1126/science.1232542

[40] Flynn SM, Dhir S, Herka K, Doyle C, Melidis L, Simeone A, Hui WWI, Araujo Tavares RDC, Schoenfelder S, Tannahill D, Balasubramanian S. Improved simultaneous mapping of epigenetic features and 3D chromatin structure via ViCAR. Genome Biol. 2024;25:237. 39227991 10.1186/s13059-024-03377-6 PMC11370281

[41] Bedrat A, Lacroix L, Mergny J-L. Re-evaluation of G-quadruplex propensity with G4Hunter. Nucleic Acids Res. 2016;44:1746–59. 26792894 10.1093/nar/gkw006 PMC4770238

[42] Jarman AP, Wood WG, Sharpe JA, Gourdon G, Ayyub H, Higgs DR. Characterization of the major regulatory element upstream of the human a-globin gene cluster. Mol Cell Biol. 1991;11:4679–89. 1875946 10.1128/mcb.11.9.4679-4689.1991 PMC361359

[43] Ney PA, Sorrentino BP, Lowrey CH, Nienhuis AW. Inducibility of the HS II enhancer depends on binding of an erythroid specific nuclear protein. Nucleic Acids Res. 1990;18:6011–7. 2235483 10.1093/nar/18.20.6011 PMC332398

[44] Elnitski L, Miller W, Hardison R. Conserved E boxes function as part of the enhancer in hypersensitive site 2 of the β-globin locus control region. J Biol Chem. 1997;272:369–78. 8995271 10.1074/jbc.272.1.369

[45] Chambers VS, Marsico G, Boutell JM, Di Antonio M, Smith GP, Balasubramanian S. High-throughput sequencing of DNA G-quadruplex structures in the human genome. Nat Biotechnol. 2015;33:877–81. 26192317 10.1038/nbt.3295

[46] Kypr J, Kejnovská I, Renčiuk D, Vorlíčková M. Circular dichroism and conformational polymorphism of DNA. Nucleic Acids Res. 2009;37:1713–25. 19190094 10.1093/nar/gkp026 PMC2665218

[47] Giraldo R, Suzuki M, Chapman L, Rhodes D. Promotion of parallel DNA quadruplexes by a yeast telomere binding protein: a circular dichroism study. Proc Natl Acad Sci. 1994;91:7658–62. 8052638 10.1073/pnas.91.16.7658 PMC44461

[48] Hardin CC, Watson T, Corregan M, Bailey C. Cation-dependent transition between the quadruplex and Watson-Crick hairpin forms of d(CGCG3GCG). Biochemistry. 1992;31:833–41. 1731941 10.1021/bi00118a028

[49] Mergny JL. Thermal difference spectra: a specific signature for nucleic acid structures. Nucleic Acids Res. 2005;33:e138–e138. 16157860 10.1093/nar/gni134 PMC1201377

[50] Mergny JL, Lacroix L. UV melting of G‐quadruplexes. Curr Protoc Nucleic Acid Chem. 2009;37:17-. 10.1002/0471142700.nc1701s37 19488970

[51] Hu JH, Navas P, Cao H, Stamatoyannopoulos G, Song C-Z. Systematic RNAi studies on the role of Sp/KLF factors in globin gene expression and erythroid differentiation. J Mol Biol. 2007;366:1064–73. 17224162 10.1016/j.jmb.2006.12.047 PMC1907364

[52] Pondel MD, Murphy S, Pearson L, Craddock C, Proudfoot NJ. Sp1 functions in a chromatin-dependent manner to augment human alpha-globin promoter activity. Proc Natl Acad Sci. 1995;92:7237–41. 7638173 10.1073/pnas.92.16.7237 PMC41314

[53] Raiber EA, Kranaster R, Lam E, Nikan M, Balasubramanian S. A non-canonical DNA structure is a binding motif for the transcription factor SP1 in vitro. Nucleic Acids Res. 2012;40:1499–508. 22021377 10.1093/nar/gkr882 PMC3287196

[54] Concordet J-P, Haeussler M. CRISPOR: intuitive guide selection for CRISPR/Cas9 genome editing experiments and screens. Nucleic Acids Res. 2018;46:W242–5. 29762716 10.1093/nar/gky354 PMC6030908

[55] Meers MP, Tenenbaum D, Henikoff S. Peak calling by sparse enrichment analysis for CUT&RUN chromatin profiling. Epigenetics Chromatin. 2019;12:1–11. 31300027 10.1186/s13072-019-0287-4 PMC6624997

[56] Phan AT, Modi YS, Patel DJ. Propeller-type parallel-stranded G-quadruplexes in the human c-myc promoter. J Am Chem Soc. 2004;126:8710–6. 15250723 10.1021/ja048805k PMC4692381

[57] Hu G, Schones DE, Cui K, Ybarra R, Northrup D, Tang Q, Gattinoni L, Restifo NP, Huang S, Zhao K. Regulation of nucleosome landscape and transcription factor targeting at tissue-specific enhancers by BRG1. Genome Res. 2011;21:1650–8. 21795385 10.1101/gr.121145.111 PMC3202282

[58] Schick S, Grosche S, Kohl KE, Drpic D, Jaeger MG, Marella NC, Imrichova H, Lin J-MG, Hofstätter G, Schuster M, et al. Acute BAF perturbation causes immediate changes in chromatin accessibility. Nat Genet. 2021;53:269–78. 33558760 10.1038/s41588-021-00777-3 PMC7614082

[59] Iurlaro M, Stadler MB, Masoni F, Jagani Z, Galli GG, Schübeler D. Mammalian SWI/SNF continuously restores local accessibility to chromatin. Nat Genet. 2021;53:279–87. 33558757 10.1038/s41588-020-00768-w

[60] Esnault C, Magat T, Zine El Aabidine A, Garcia-Oliver E, Cucchiarini A, Bouchouika S, Lleres D, Goerke L, Luo Y, Verga D, et al. G4access identifies G-quadruplexes and their associations with open chromatin and imprinting control regions. Nat Genet. 2023;55(8):1359–69. 37400615 10.1038/s41588-023-01437-4

[61] Henikoff S, Henikoff JG, Kaya-Okur HS, Ahmad K. Efficient chromatin accessibility mapping in situ by nucleosome-tethered tagmentation. eLife. 2020;9:e63274. 33191916 10.7554/eLife.63274 PMC7721439

[62] Barski A, Cuddapah S, Cui K, Roh T-Y, Schones DE, Wang Z, Wei G, Chepelev I, Zhao K. High-resolution profiling of histone methylations in the human genome. Cell. 2007;129:823–37. 17512414 10.1016/j.cell.2007.05.009

[63] Heintzman ND, Stuart RK, Hon G, Fu Y, Ching CW, Hawkins RD, Barrera LO, Van Calcar S, Qu C, Ching KA, et al. Distinct and predictive chromatin signatures of transcriptional promoters and enhancers in the human genome. Nat Genet. 2007;39:311–8. 17277777 10.1038/ng1966

[64] Krijger PHL, Geeven G, Bianchi V, Hilvering CRE, de Laat W. 4C-seq from beginning to end: A detailed protocol for sample preparation and data analysis. Methods. 2020;170:17–32. 31351925 10.1016/j.ymeth.2019.07.014

[65] Molete JM, Petrykowska H, Bouhassira EE, Feng Y-Q, Miller W, Hardison RC. Sequences flanking hypersensitive sites of the β-globin locus control region are required for synergistic enhancement. Mol Cell Biol. 2001;21:2969–80. 11287603 10.1128/MCB.21.9.2969-2980.2001 PMC86926

[66] Kim S, Kim YW, Shim SH, Kim CG, Kim A. Chromatin structure of the LCR in the human β-globin locus transcribing the adult δ- and β-globin genes. Int J Biochem Cell Biol. 2012;44:505–13. 22178075 10.1016/j.biocel.2011.12.001

[67] Vaughn JP, Creacy SD, Routh ED, Joyner-Butt C, Jenkins GS, Pauli S, Nagamine Y, Akman SA. The DEXH protein product of the DHX36 gene is the major source of tetramolecular quadruplex G4-DNA resolving activity in HeLa cell lysates. J Biol Chem. 2005;280:38117–20. 16150737 10.1074/jbc.C500348200

[68] Law MJ, Lower KM, Voon HPJ, Hughes JR, Garrick D, Viprakasit V, Mitson M, De Gobbi M, Marra M, Morris A, et al. ATR-X syndrome protein targets tandem repeats and influences allele-specific expression in a size-dependent manner. Cell. 2010;143:367–78. 21029860 10.1016/j.cell.2010.09.023

[69] Dai Y, Teng X, Zhang Q, Hou H, Li J. Advances and challenges in identifying and characterizing G-quadruplex–protein interactions. Trends Biochem Sci. 2023;48:894–909. 37422364 10.1016/j.tibs.2023.06.007

[70] Makowski MM, Gräwe C, Foster BM, Nguyen NV, Bartke T, Vermeulen M. Global profiling of protein–DNA and protein–nucleosome binding affinities using quantitative mass spectrometry. Nat Commun. 2018;9:1653. 29695722 10.1038/s41467-018-04084-0 PMC5916898

[71] Li W, Notani D, Rosenfeld MG. Enhancers as non-coding RNA transcription units: recent insights and future perspectives. Nat Rev Genet. 2016;17:207–23. 26948815 10.1038/nrg.2016.4

[72] Harrison LJ, Bose D. Enhancer RNAs step forward: new insights into enhancer function. Development. 2022;149:dev200398. 36039999 10.1242/dev.200398 PMC9481971

[73] Barshad G, Lewis JJ, Chivu AG, Abuhashem A, Krietenstein N, Rice EJ, Ma Y, Wang Z, Rando OJ, Hadjantonakis AK, Danko CG. RNA polymerase II dynamics shape enhancer–promoter interactions. Nat Genet. 2023;55(8):1370–80. 37430091 10.1038/s41588-023-01442-7 PMC10714922

[74] Zhang S, Übelmesser N, Barbieri M, Papantonis A. Enhancer–promoter contact formation requires RNAPII and antagonizes loop extrusion. Nat Genet. 2023;55:832–40. 37012454 10.1038/s41588-023-01364-4

[75] Vo N, Goodman RH. CREB-binding protein and p300 in transcriptional regulation. J Biol Chem. 2001;276:13505–8. 11279224 10.1074/jbc.R000025200

[76] Catarino RR, Stark A. Assessing sufficiency and necessity of enhancer activities for gene expression and the mechanisms of transcription activation. Genes Dev. 2018;32:202–23. 29491135 10.1101/gad.310367.117 PMC5859963

[77] Narita T, Ito S, Higashijima Y, Chu WK, Neumann K, Walter J, Satpathy S, Liebner T, Hamilton WB, Maskey E, et al. Enhancers are activated by p300/CBP activity-dependent PIC assembly, RNAPII recruitment, and pause release. Mol Cell. 2021;81:2166-2182.e2166. 33765415 10.1016/j.molcel.2021.03.008

[78] Fujiwara T, O’Geen H, Keles S, Blahnik K, Linnemann AK, Kang Y-A, Choi K, Farnham PJ, Bresnick EH. Discovering hematopoietic mechanisms through genome-wide analysis of GATA factor chromatin occupancy. Mol Cell. 2009;36:667–81. 19941826 10.1016/j.molcel.2009.11.001 PMC2784893

[79] Bernecky C, Herzog F, Baumeister W, Plitzko JM, Cramer P. Structure of transcribing mammalian RNA polymerase II. Nature. 2016;529:551–4. 26789250 10.1038/nature16482

[80] Cramer P, Bushnell DA, Kornberg RD. Structural basis of transcription: RNA polymerase II at 2.8 Ångstrom resolution. Science. 2001;292:1863–76. 11313498 10.1126/science.1059493

[81] Varshney D, Spiegel J, Zyner K, Tannahill D, Balasubramanian S. The regulation and functions of DNA and RNA G-quadruplexes. Nat Rev Mol Cell Biol. 2020;21:459–74. 32313204 10.1038/s41580-020-0236-x PMC7115845

[82] Zhang X, Spiegel J, Martínez Cuesta S, Adhikari S, Balasubramanian S. Chemical profiling of DNA G-quadruplex-interacting proteins in live cells. Nat Chem. 2021;13:626–33. 34183817 10.1038/s41557-021-00736-9 PMC8245323

[83] Geeven G, Teunissen H, de Laat W, de Wit E. peakC: a flexible, non-parametric peak calling package for 4C and capture-C data. Nucleic Acids Res. 2018;46:e91–e91. 29800273 10.1093/nar/gky443 PMC6125690

[84] Doyle C, Herka K, Flynn SM, Melidis L, Dhir S, Tannahill D, Schoenfelder S, Balasubramanian S. DNA G-quadruplex structures act as functional elements in α- and β-globin enhancers. GSE267666. Gene Expression Omnibus. 2025. https://www.ncbi.nlm.nih.gov/geo/query/acc.cgi?acc=GSE267666. 10.1186/s13059-025-03627-1 PMC12139101 40468392

[85] Perez-Riverol Y, Bandla C, Kundu DJ, Kamatchinathan S, Bai J, Hewapathirana S, John NS, Prakash A, Walzer M, Wang S, Vizcaíno JA. The PRIDE database at 20 years: 2025 update. Nucleic Acids Res. 2025;53(D1):D543–53. 39494541 10.1093/nar/gkae1011 PMC11701690

[86] ENCODE Project Consortium. ENCSR858MPS. 10.17989/2FENCSR858MPS.

[87] ENCODE Project Consortium. ENCSR000EGM. 10.17989/2FENCSR000EGM.

[88] ENCODE Project Consortium. ENCSR000EWF. 10.17989/2FENCSR000EWF.

[89] Doyle C, Herka K, Flynn SM, Melidis L, Dhir S, Tannahill D, Schoenfelder S, Balasubramanian S. DNA G-quadruplex structures act as functional elements in α- and β-globin enhancers. Enhancer_G4s. Github. 2025. https://github.com/sblab-informatics/Enhancer_G4s. 10.1186/s13059-025-03627-1 PMC12139101 40468392

[90] Doyle C, Herka K, Flynn SM, Melidis L, Dhir S, Tannahill D, Schoenfelder S, Balasubramanian S. DNA G-quadruplex structures act as functional elements in α- and β-globin enhancers. Enhancer_G4s. 2025. Zenodo.10.5281/zenodo.15391109. 10.1186/s13059-025-03627-1 PMC12139101 40468392

