# Supplementary material for: DNA G-quadruplex structures act as functional elements in α- and β-globin enhancers
Source: Genome Biol. 2025 Jun 4;26:155. doi: 10.1186/s13059-025-03627-1 (PMC12139101; doi:10.1186/s13059-025-03627-1)
Supplement: Supplementary file 1 — Additional file 1: Fig. S1-S7. Supplementary figures. [file 13059_2025_3627_MOESM1_ESM.docx]

**DNA G-quadruplex structures act as functional elements in α-** **and β-globin enhancers**

**Supplementary information figures S1-S7**

**Fig. S1. Biophysical characterisation of HS-40 and HS2 enhancer G4s (related to Figure 2). A)** UV thermal difference spectra of HS-40 and HS2 putative G4 forming sequences. **B)** UV melting analysis of HS-40 and HS2 putative G4 forming sequences and their mutated counterparts.

**Fig. S2. SP1 does not exhibit binding potential for HS-40 or HS2 WT or mutant G4 motifs in duplex DNA (related to Figure 2). A)** Sanger sequencing traces of G4 edited HS-40 and HS2 loci in αMut1, αMut2 and βMut1, βMut2 cells respectively, inserted sequences are highlighted in blue. **B)** Chemiluminescence images of oligonucleotide pulldown samples probed with anti-SP1 antibody on the Simple Western platform (Protein Simple). Lysate: 10% of nuclear lysate used as input for pulldown. Beads: bead only control with no bound oligonucleotide probe, G4: single strand αWT/βWT G4 oligonucleotide. Duplex: G4 and αMut/βMut sequences in duplex DNA with complementary strand. Consensus duplex: SP1 consensus motif 5’-GGGGCGGGG-3’ in duplex DNA.

**Fig. S3. Cellular characterisation of HS-40 and HS2 enhancer G4s (related to Figure 2). A)** IGV browser view of G4 signal by BG4 CUT&Tag at HS-40 or HS2 enhancers in additional CRISPR edited clones. **B)** IGV browser view of G4 signal by BG4 CUT&Tag at two G4 unedited positive control sites. These are the same samples shown in Supplementary Fig. 3A. **C)** IGV browser view of G4 signal by CUT&Tag with BG4 or SG4 at two G4 unedited positive control sites (top and bottom panel) in wild type (αWT), HS-40 G4 mutated (αMut) and HS-40 G4 restored (αRes) cells. These are the same samples as shown in Fig. 2E. **D)** IGV browser view of G4 signal by CUT&Tag with BG4 or SG4 at two G4 unedited positive control sites (top and bottom panel) in wild type (βWT) and HS2 G4 mutated (βMut) cells. These are the same samples as shown in Fig. 2G. **E)** Bar plots of cpm normalised read counts of CUTAC signal across the HS-40 G4 enhancer motif and at a KRAS control site in wild type (WT), G4 mutated (αMut, βMut) and G4 restored (αRes) cells. Error bars indicate the standard deviation (SD), *P-value < 0.05, n.s is not significant. **F)** Bar plots of cpm normalised read counts of CUTAC signal across the HS2 enhancer G4 motif and at a SDF4 control site in wild type (WT), G4 mutated (βMut) cells. Error bars represent the standard deviation (SD), **P-value < 0.01, n.s is not significant. **G)** IGV browser view of CUTAC signal at unedited control sites (left and right panel) in wild type (αWT, βWT), G4 mutated (αMut, βMut) and G4 restored (αRes) cells. These are the same samples as shown in Fig. 2I,J.

**Fig. S4. Genotyping of G4 restored αMut cell lines (related to Figure 2).** Sanger sequencing traces of the G4 restored in HS-40 locus in four single clone cell populations that were derived from CRISPR editing of αMut1 cells, inserted sequences are highlighted in blue.

**Fig. S5. Consistent 3D chromatin interactions across different G4 mutated cell clones (related to Figure 3).** **A)** 4C signal from viewpoint of the α-globin HS-40 enhancer in wild type (WT, blue) and G4 mutated (αMut 2 ,red) cells. WT sample is the same data as shown in Figure 3C. **B)** Table of log2FC values and P-values for *HBA1* expression in αMut1 or αMut2 compared to G4-restored (αRes1-4) cell lines. **C)** 4C signal from the viewpoint of the β-globin HS2 and **D)** HS3 enhancers in wild type (WT, blue) and G4-mutated (βMut 2, red) cells. Overlap is indicated in grey. **E)** IGV browser view of H3K27ac signal by CUT&Tag across the β-globin locus in wild type (WT) and G4 mutated (βMut 2) cells.

**Fig. S6. HS3 deletion in βMut cells (related to Figure 3).** **A)** Sanger sequencing traces of breakpoints at the HS3 enhancer in wild type (WT HS3Δ) and G4 mutated plus HS3 enhancer deleted (βMut HS3Δ) cell lines showing 5 base pair difference between WT HS3Δ and βMut HS3Δ. **B)** Table of log2FC values and P-values for *HBE1* expression in βMut1 HS3Δ compared to WT HS3Δ cells. **C)** IGV browser view of H3K27ac and H3K4me1 signal across the β-globin locus by CUT&Tag in wild type (WT), enhancer HS3 deleted (HS3Δ) and G4 mutated plus HS3 enhancer deleted (βMut HS3Δ) cells. Grey shading indicates the HS2 enhancer, purple shading indicates the HS3 enhancer. **D)** Bar plots of cpm normalised read counts of H3K27ac and H3K4me1 by CUT&Tag at the β-globin HS2 enhancer in wild type (WT) and G4 mutated (βMut) cells (n=4). Error bars represent the standard deviation (SD), *P-value < 0.05 and **P-value < 0.01.

**Fig. S7. Architectural proteins do not bind G4s structure *in vitro* or in cells (related to figure 4). A)** Volcano plot highlighting a range of chromatin architectural proteins and proposed chromatin looping factors which are not significantly differentially captured by oligonucleotides for wild type (αWT) or mutated HS-40 α-globin G4s. The red line indicates -log10 of the adjusted P-value of 0.05. **B)** Experimentally validated G4-interacting proteins significantly differentially enriched by affinity capture for αMut vs WT. **C-D)** IGV browser view of CTCF (ENCFF322EGW) and YY1 (ENCFF577NSA) ChIP-seq data from ENCODE across the α- and β-globin loci, HS-40 is indicated by pink shading, HBA1 is indicated by blue shading and HS2 by grey shading. **E)** Binding curve determined by ELISA assay for the POLR2C subunit of the RNAPII complex for HS-40 (αWT) and HS2 (βWT) enhancer G4 oligonucleotides and their mutant controls (αMut, βMut).
